# Pancreatic Carcinoma: the Disease that Kills

**DOI:** 10.14740/wjon954w

**Published:** 2016-04-03

**Authors:** Serbeze Kabashi, Kreshnike Dedushi, Naser Ramadani, Sefedin Mucaj, Asrtrit Hoxhaj, Naim Jerliu

**Affiliations:** a Faculty of Medicine, Pristine University, Pristine City 10000, Kosovo; b Department of Radiology, Diagnostic Centre, UCCK, Pristine City 10000, Kosovo; c National Institute of Public Health of Kosovo, Pristine City 10000, Kosovo; d Hospital Hygeia, Tirana City 10004, Albania

**Keywords:** Adenocarcinoma, ECHO, Multidetector computerized tomography, Hepatic metastasis, Contrast, AFP, CEA, CA19-9

## Abstract

The purpose of this case report is to demonstrate the clinical symptoms and laboratory changes that have occurred very late and were very few in number even the imaging studies performed at that time showed an intensive local tumor growth associated with the wide infiltration of the both adjacent and distant upper abdominal structures. A 71-year-old male patient who was a chronic alcohol abuser and ex smoker (quit smoking 8 years earlier) presented with symptoms of mild pain on epigastric region that irradiated toward the back and significant weight loss. The initial ultrasonography (US) examination was performed, followed by the lab tests and multidetector computed tomography (MDCT) examination. The diagnostic studies confirmed the presence of the pancreatic’s body mass. The ordered laparoscopic evaluation established definitive diagnosis. Initial US examination showed heterogeneous pseudo-cystic changes and slight edema of the pancreatic parenchyma associated with the multiple oval hyperechogenic lesions of liver - the signs highly suggestive of secondary metastatic deposits. The other imaging findings that were obtained with the use of the MDCT confirmed the presence of an expansive primary process of the body of the pancreas associated with the secondary metastatic changes in liver. In addition, the consecutive lymphadenopathy was revealed along hepatoduodenal ligament, retropancreatic region and intraperitoneal compartment. Tumor markers resulted with the high values of the AFP of 2.3, CA19-9 of 423.0 U/mL, and CEA of 219.0 ng/mL. The specimen of the tumor tissue taken during laparoscopic biopsy was sent for histologic examination and the final result was “metastatic adenocarcinoma of pancreas”. Pancreatic body carcinoma has always been associated with poor prognosis because diagnosis is made at the advanced stage of the disease. Therefore, poor prognosis might be improved if early diagnosis could be made. Recent researches confirmed genetic predisposition for this disease at certain group of patients and this “high risk” group has to be followed up with regular imaging studies and lab analysis.

## Introduction

Pancreatic cancer is the fourth leading cause of cancer deaths among men and women, being responsible for 6% of all cancer-related deaths. Approximately 75% of all pancreatic carcinomas occur within the head or neck of the pancreas, 15-20% occur in the body of the pancreas, and 5-10% occur in the tail [1, 2].

Pancreatic cancer is sometimes called a “silent cancer” because there are generally no symptoms in the early stages. Early pancreatic cancer symptoms are often vague and unrecognized and can be often dismissed by patients and doctors. The longer the time without established diagnosis, the less the options for potentially lifesaving surgery [3, 4].

### Objectives

The purpose of this case report is to demonstrate the clinical symptoms and laboratory changes that have occurred very late, even the tumor was very large and has caused multiple liver metastasis and lymphadenopathy along hepatoduodenal ligament, retropancreatic and intraperitoneal regions at the moment of diagnostication.

Initially, pancreatic cancer tends to be silent and painless as it grows. By the time, due to the continuous growth, the size of the tumor itself starts to cause clinical symptoms dependent on local changes. Pancreatic cancer generally grows outside the pancreas. At this point, symptoms depend on the cancer’s location within the pancreas: 1) Pancreatic cancer localized on the pancreatic head tends to cause symptoms such as weight loss, jaundice (yellow skin), dark urine, light color of the stool, itching, nausea, vomiting, abdominal pain and back pain; 2) Pancreatic cancer in the body or tail of the pancreas usually causes belly and/or back pain as well as the weight loss.

In general, symptoms appear earlier if cancers occur in the head of the pancreas, compared to those localized in the body and tail.

## Case Report

The patient was a 71-year-old male, who was a chronic alcohol abuser and ex chronic smoker (quit smoking 8 years ago). On admission at gastroenterologist, the patient complained of epigastric pain with back irradiation and weight loss.

**Figure 1:**
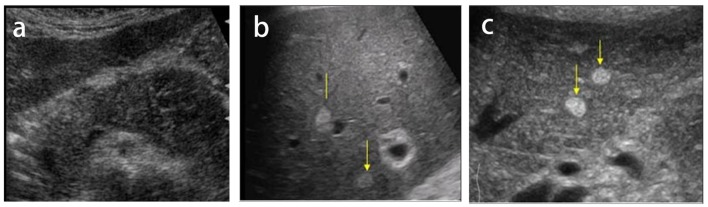
Ultrasound images (a, b, c) revealed edematous and heterogeneous pseudo-cystic changes on the body of pancreatic parenchyma (Department of Radiology in the University Clinical Center of Kosovo, Pristine).

Patient was appointed in our clinic for routine abdominal ultrasound examination that revealed edematous and heterogeneous pseudo-cystic changes on the body of the pancreatic parenchyma. US examination also showed multiple oval hyperechogenic lesions with sonographic features of metastasis (Fig. 1a, b, c).

**Figure 2:**
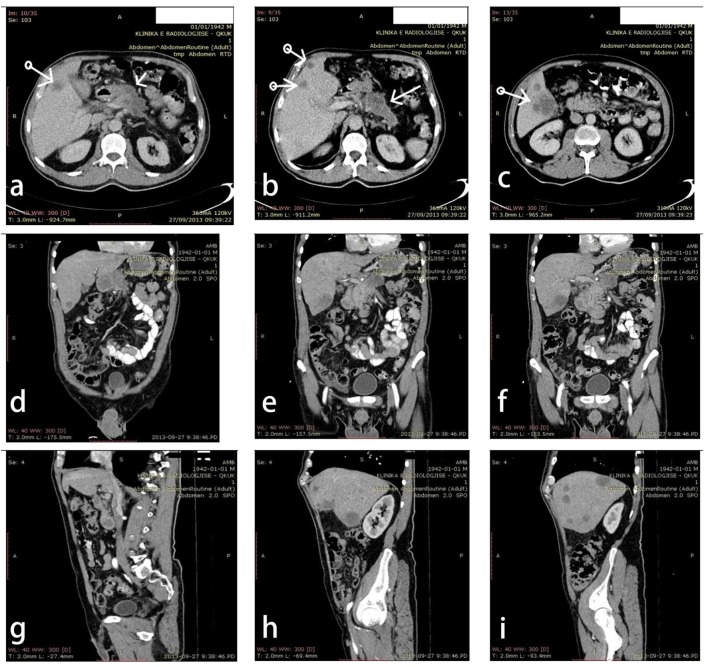
Multidetector computed tomography: axial plane (a, b, c); coronal plane (d, e, f); sagittal plane (g, h, i); results are highly suggestive of primary neoplastic process on the body of the pancreas with multiple liver metastasis and lymphadenopathy along hepatoduodenal ligament, and retropancreatic and intraperitoneal regions.

Laboratory analysis (tumor markers) results showed high values of AFP of 2.3, CA19-9 of 423.0 U/mL, and CEA of 219.0 ng/mL. Examination with multidetector computed tomography (MDCT) was requested, and after initial non-contrast-enhanced computed tomography (NECT), the examination with use of contrast-enhanced multidetector computed tomography (CECT) was performed. Axial (Fig. 2a, b, c), coronal (Fig. 2d, e, f)) and sagittal (Fig. 2g, h, i) reconstructions showed enlargement of the body of the pancreas due to the presence of the multicameral pseudocystic formation. The transverse dimensions of the mass were 90 × 30 mm. Peripheral and interseptal central contrast enhancement was evident. Liver presented multifocal parenchymal hypodens lesions, with moderate contrast enhancement and maximal transverse dimension of 40 mm. A significantly enlarged lymph node was seen within hepatoduodenal ligament while small lymph nodes were detected in retropancreatic and intraperitoneal regions. MDCT results were highly suggestive of primary neoplastic process localized on the body of the pancreas with multiple liver metastasis and lymphadenopathy along hepatoduodenal ligament, retropancreatic and intraperitoneal regions.

Laparoscopic biopsy was done and specimen for histopathology was taken. The final result was “metastatic adenocarcinoma originated from pancreas with liver metastasis”.

**Figure 3:**
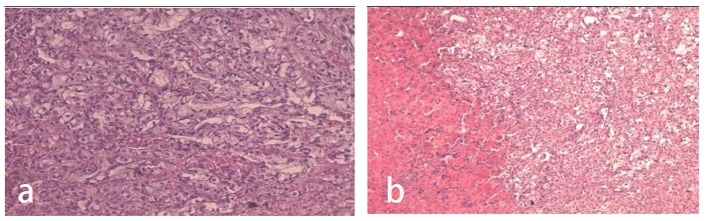
Histopathology findings (a, b) showing the erythrocyte domination and multiple clusters of malign epithelial cells. In some microscopic fields, tumor cells cause obliteration of the vascular lumens (Institute of Pathology in the University Clinical Center of Kosovo, Pristine).

Histopathology samples showed red blood cells domination and presence of many clusters of malign epithelial cells. In some microscopic fields, tumor cells caused obliterations of the vascular lumens (Fig. 3a, b).

Based on the imaging findings and histopathology diagnosis, the tumor was classified as “stage IVa, T3N1M1 metastatic adenocarcinoma of the pancreas” - inoperable case.

Patient underwent chemotherapy and palliative care according to current protocols. The treatment was clearly unsuccessful and after 6 weeks patient passed away.

## Discussion

The incidence rate for pancreatic head cancer has remained at 5.6% per 100,000, whereas the rate for pancreatic body/tail cancers has increased up to 46% between 1973 and 2002. The 3-year survival rate has also increased (even slightly) for both groups [5-7]. Unfortunately, our patient could not reach such a survival period.

“Silent disease” that had very few clinical symptoms with late occurrence resulted with poor prognosis in our patient. The initial symptoms that were noticed were mild epigastric pain with back irradiation and gradual weight loss. First clinical opinions related subjective complaints to patient’s addictions and age - the epigastric pain was considered to be result of chronic alcohol abuse and specific food diet that patient applied lately and was interpreted as dyspepsia. Furthermore, the food diet was considered as responsible for gradual weight loss. Back pain was interpreted itself as a common symptom frequently experienced in elderly patients with spine degenerative diseases.

Abovementioned symptoms that occurred at our patient are not highly specific for pancreatic cancer and can be found as common complaints among many diseases that have mostly abdominal origin [6, 7].

The “clue” that was needed to establish more accurate diagnosis was employment of diagnostic imaging modalities and lab studies related with detection of tumor markers. The final result was achieved after laparoscopic biopsy and histology examination.

### Conclusion

The clinical awareness regarding this disease has to be increased because the symptoms that occur are very discrete and very late and appropriate diagnostic steps have to be taken as soon as possible in order to exclude or confirm pancreatic cancer. Having in consideration the poor health care conditions in our country as well as deficient access on diagnostic procedures, it is expected that patients with pancreatic cancer will be diagnosed at the late stages of the disease, and as a result, the prognosis for longer term survival and the life quality is expected to be low.
